# Relationship Between Illness Representations, Psychosocial Adjustment, and Treatment Outcomes in Mental Disorders: A Mini Review

**DOI:** 10.3389/fpsyg.2020.01167

**Published:** 2020-06-12

**Authors:** Priscillia Averous, Elodie Charbonnier, Lionel Dany

**Affiliations:** ^1^Aix Marseille Univ, LPS, Aix-en-Provence, France; ^2^UNIV. NIMES, EA 7352 CHROME, Nîmes, France; ^3^APHM, Timone, Service d’Oncologie Médicale, Marseille, France

**Keywords:** illness perceptions, self-regulation model, outcome, treatment, mental health

## Abstract

Understanding and improving the psychosocial adjustments (e.g., quality of life, depression) and treatment outcomes (e.g., adherence, beliefs about treatments) of people with mental disorders are major health issues. The self-regulation model (SRM) postulates that illness representations play a central role on adjustment and treatment of people with physical illnesses. Recently, the SRM has been used with people with mental disorders. However, the manifestations of somatic and psychiatric disorders can be very different. Therefore, the use of SRM in the field of mental health is very complex. This difficulty, as well as the growing interest for illness representations in the field of mental health, justifies the utility to conduct a review on this topic. The current review shows that illness representations are related to psychosocial adjustment and/or treatment outcomes for people with various mental disorders [e.g., psychotic disorders, mood disorder, posttraumatic stress disorder (PTSD), attention deficit hyperactivity disorder (ADHD)]. However, some limitations to the applicability of SRM to mental disorders have been highlighted. These limitations should be considered in future studies.

## Introduction

Psychosocial adjustment of people with mental disorders is severely diminished, in particular, they live 10 years less than the general population (Walker et al., 2015), with a lower quality of life (Alonso et al., 2004; Fleury et al., 2013) and a higher level of disability (Alonso et al., 2004). To manage these disorders, the emphasis is on medication and/or psychosocial interventions. However, for many disorders, a low level of adherence to treatment has been observed (Lacro et al., 2002; García et al., 2016; Ho et al., 2016). Poor adherence hinders psychosocial adjustment, diminishes the effect of treatment, reduces quality of life, and increases the cost to the health care system (World Health Organization, 2003). Therefore, understanding the processes involved in the psychosocial adjustment and adherence of people with mental disorders is a major health issue.

In physical illnesses, many studies have highlighted the central role of illness representations on psychosocial adjustment (for reviews and/or meta-analysis, see Hagger and Orbell, 2003; Foxwell et al., 2013; Al-Smadi et al., 2016; Hagger et al., 2017) and adherence (for a meta-analysis, see Brandes and Mullan, 2014). According to the self-regulation model (SRM; Leventhal et al., 1984), illness representations can be divided into six dimensions: *timeline* (considering whether the illness is acute, chronic, or cyclical); *consequences* (assessing its impact on life, including physical, emotional, social, and economic outcomes); *cure/control*, which can be divided into personal control (beliefs about personal abilities to control the illness), and treatment control (beliefs about the treatment’s effectiveness in curing or managing the illness); *identity* (overall comprehensibility of the illness); *emotional representations* (emotional impact or emotional response to the illness); and *cause* (factors believed to be responsible for the illness or condition).

The SRM was originally created to try to understand what people think and how they cope with a concrete physical illness that can potentially affect their mental health. More recently, the SRM has been used with people with mental disorders. However, the manifestations of somatic and psychiatric disorders are very different. For example, the symptoms of a mental disorder can change an individual’s cognitions, behaviors, and emotions. Therefore, the use of SRM in the field of mental health is very complex. To date, only two reviews (Lobban et al., 2003; Baines and Wittkowski, 2013) have examined the applicability of SRM for people with mental disorders. However, the most recent studies included in these reviews are from 2002 for one (Lobban et al., 2003) and 2011 for the other (Baines and Wittkowski, 2013). The growing interest for illness representations in the field of mental health justifies the utility to conduct more recent review. This mini review intends to answer the following question: what are the links between representations of illness, psychosocial adjustment, and treatment outcomes in individuals with a mental disorder. Additional information on each study included (e.g., population, design, and results) are available in the Supplementary Material.

## Method

In order to identify the articles answering our question, we used the following keywords: *self-regulation model* OR *illness perception ^∗^* OR *illness representation ^∗^* OR AND *mental disorder ^∗^* OR *mental health*. We conducted electronic searches on PsycINFO, PsycARTICLES, Web of Science, and PubMed to identify studies published between 1980 and 2019. The following inclusion criteria were applied to the articles: (1) Published empirical study or research report examining illness representation in the field of mental health, (2) Dealing with psychosocial adjustment and/or treatment outcomes, and (3) Article written in English or French and published in a peer-reviewed journal. In this review, 47 articles were selected.

## Results

### Illness Representations and Psychosocial Adjustment of People With Mental Disorders

Twelve studies have explored the relationship between psychosocial adjustment and illness representations in people with psychotic disorders (Lobban et al., 2004, 2005, 2006; Fialko et al., 2006; Watson et al., 2006; Stainsby et al., 2010; Cavelti et al., 2012a,b; Moriarty et al., 2012; Theodore et al., 2012; Gómez-de-Regil et al., 2014; Maguire et al., 2016). Poor psychosocial adjustment (e.g., high symptoms, low quality of life) was related to high identity (Lobban et al., 2005; Watson et al., 2006), high chronicity (Lobban et al., 2005; Fialko et al., 2006; Watson et al., 2006), high cyclicity (Lobban et al., 2005), high consequences (Lobban et al., 2005; Fialko et al., 2006; Watson et al., 2006; Stainsby et al., 2010; Theodore et al., 2012), and high emotional representations (Lobban et al., 2005; Cavelti et al., 2012b; Theodore et al., 2012; Gómez-de-Regil et al., 2014). Conversely, good psychosocial adjustment (e.g., high subjective well-being, high insight) was associated with high levels of personal control (Lobban et al., 2005), treatment control (Lobban et al., 2005; Cavelti et al., 2012b; Theodore et al., 2012), cure/control (Fialko et al., 2006; Watson et al., 2006), and coherence (Lobban et al., 2005; Stainsby et al., 2010; Cavelti et al., 2012b). A high level of perceived consequences was one of the strongest predictors of poor psychosocial adjustment (e.g., high symptoms, low functioning) (Lobban et al., 2004). Moreover, low levels of personal control and coherence and high levels of consequence, identity, concern, and emotional representation were predicted by self-rated mental health rated as being “poor and/or fair” (in comparison with excellent). Higher chronicity was predicted by self-rated mental health rated as being “very good” (in comparison with excellent; Maguire et al., 2016). Treatment control was helpful to explain the quality of life (Theodore et al., 2012). Moreover, cognitive and emotional representations mediated the link between residual symptoms and quality of life (Gómez-de-Regil et al., 2014). One study highlighted that the association between insight and depressive symptoms was mediated by chronicity and consequences (Cavelti et al., 2012a). In addition, illness representations were not related to the level of activity (Moriarty et al., 2012). Finally, for people with a high level of expressed emotion, a significant gap was observed between illness representations of relatives and patients (Lobban et al., 2006).

Four studies have explored the relationship between psychosocial adjustment and illness representations in people with a bipolar disorder (Lobban et al., 2013; Peay et al., 2013, 2014; Dodd et al., 2017). Good recovery was associated with high levels of treatment and personal control and low levels of perceived consequences, emotional representations, identity, and self-blame (Dodd et al., 2017). Identity, consequences, and personal concern had an effect on time to relapse (Lobban et al., 2013). Personal control was negatively related to depression (Lobban et al., 2013). The relationship between representation of illness severity and adaptation was mediated by coping (Peay et al., 2013). Finally, severity perceived was not linked with parents’ ability to cope with the risk of mood disorders in children (Peay et al., 2014).

Three studies have explored the relationship between psychosocial adjustment and illness representations in people with mixed mental disorders (Broadbent et al., 2008; Ward and Heidrich, 2009; Chan and Mak, 2016). A low level of functioning was related to a low level of control and high levels of identity, concern, emotional representations, timeline, and consequences (Broadbent et al., 2008). High personal control was a predictor of good recovery (Chan and Mak, 2016). Finally, stigma was negatively associated with identity (Ward and Heidrich, 2009) and personal control (Chan and Mak, 2016).

Three studies have explored the relationship between psychosocial adjustment and illness representations in people with depression (Kelly et al., 2007; Cabassa et al., 2008; Lu et al., 2014). Poor psychosocial adjustment (e.g., high symptoms) was associated with high levels of chronicity (Cabassa et al., 2008; Lu et al., 2014), identity, consequences, and emotional representations and low levels of control and coherence (Lu et al., 2014). High emotional representations promoted the use of maladaptive coping. A high level of consequences was related to a low level of problem-solving strategies (Kelly et al., 2007). Finally, the association between illness representations and emotional outcomes was mediated by maladaptive ruminations (Lu et al., 2014).

Two studies have explored the relationship between psychosocial adjustment and illness representations in people with an eating disorder (Quiles Marcos et al., 2009; DeJong et al., 2012). For people with bulimia, low control was related to a high shape concern. A high level of emotional representations was associated with high levels of anxiety, dietary restraint, eating concern, and shape/weight concern. Finally, identity was positively associated with dietary restraint (DeJong et al., 2012). For people with various eating disorders, when both patients and their relatives had high personal control, patients had lower depressive and anxious symptomatology in comparison to dyads with different representations. Similarly, when both patients and their relatives had high treatment control, patients had better psychosocial adjustment than dyads with different scores (Quiles Marcos et al., 2009).

Finally, four studies have explored the relationship between psychosocial adjustment and illness representations in adolescents with a mental disorder (Munson et al., 2009; Moses, 2010, 2015; Wong et al., 2019). For adolescents with affective and disruptive disorders, a high level of the following causal attributions: trauma and social problems were related to high self-stigma (Moses, 2010). For adolescents with various mental disorders, high control and low chronicity were related to high self-esteem (Moses, 2015). For adolescents with mood disorders, high emotional representations predicted high stigma (Munson et al., 2009). For adolescents with attention deficit hyperactivity disorder (ADHD), a good quality of life was predicted by a low level of “impact” representation (i.e., consequences, identity, concern, and emotional representation) and a high level of psychological or environmental causal attributions of ADHD. In addition, a high level of minimization was predicted by high levels of timeline, personal control, coherence, and low level of consequence perceived (Wong et al., 2019).

### Illness Representations and Treatment Outcomes of People With Mental Disorders

Five studies have explored the relationship between treatment outcomes and illness representations in people with psychotic disorders (Watson et al., 2006; Rungruangsiripan et al., 2011; Beck et al., 2012; Cavelti et al., 2012b; Marcus et al., 2014). The necessity to take a treatment was related to high levels of treatment control (Beck et al., 2012; Cavelti et al., 2012b), chronicity, and cyclicity (Cavelti et al., 2012b). A high level of concern about treatment was associated with high scores of consequences (Beck et al., 2012; Cavelti et al., 2012b) and emotional representations (Cavelti et al., 2012b) and a low score of treatment control (Beck et al., 2012; Cavelti et al., 2012b). High distrust of medicine was related to high consequences and low treatment control (Beck et al., 2012). A study showed that cure/control was a predictor of the impact of cognitive and behavioral therapy (Marcus et al., 2014). Therapeutic alliance and medication side effects have impacted illness representations, which in turn, have impacted the intention to change (Rungruangsiripan et al., 2011). Finally, perceived consequences were negatively related to adherence to medication (Watson et al., 2006).

Five studies have explored the relationship between treatment outcomes and illness representations in people with a bipolar disorder (Hou et al., 2010; Oflaz et al., 2015; Averous et al., 2018; Etain et al., 2018; M’Bailara et al., 2019). Good treatment adherence was predicted by high treatment control and low emotional representation (Averous et al., 2018). Non-adherent patients had higher levels of perceived consequences and chronicity than adherent patients (Hou et al., 2010). Dropout patients had higher emotional representations and personal control, and lower consequences, than adherent patients (Oflaz et al., 2015). Two studies have shown that after educational therapies, illness representations were improved (Etain et al., 2018; M’Bailara et al., 2019).

Five studies have explored the relationship between treatment outcomes and illness representations in people with mixed mental disorders (Hunot et al., 2007; Broadbent et al., 2008; Vanheusden et al., 2009; Williams and Steer, 2011; Reich et al., 2015). High chronicity, control (personal and treatment), and coherence, and low emotional representations, were associated with a positive attitude toward treatment (Broadbent et al., 2008). High levels of control and coherence, and a low level of consequences perceived, were related to greater engagement in treatment (Williams and Steer, 2011). Perceived consequences, treatment control, and beliefs about intrapsychic causes were positively associated with health services use (Vanheusden et al., 2009). Illness representations were predictors of motivation for psychotherapy (Reich et al., 2015). Finally, one study did not find a link between illness representations and adherence (Hunot et al., 2007).

Four studies have explored the relationship between treatment outcomes and illness representations in people with depression (Aikens et al., 2008; O’Mahen et al., 2009; Houle et al., 2013; Elwy et al., 2016). Timeline was a predictor of the need to receive treatment (Aikens et al., 2008) and treatment use (medication or psychotherapy/counseling) in women with perinatal depression (O’Mahen et al., 2009). A low coherence was a predictor of a high perception of the harmfulness of medication (Aikens et al., 2008). A high level of consequences perceived was associated with a high level of commitment to psychotherapy (Houle et al., 2013). Veterans with high levels of cyclicity and control were less likely to receive guideline-concordant depression treatment (Elwy et al., 2016). The belief that depression was caused by a chemical imbalance was associated with a higher need to take treatment. The belief that depression was caused by bad luck/chance was related to a higher perception of the harmfulness of medications (Aikens et al., 2008). Social attributions (e.g., problems in the family) were positively related to commitment in psychotherapy (Houle et al., 2013).

One study explored the relationship between treatment outcomes and illness representations in veterans with posttraumatic stress disorder (PTSD) (Spoont et al., 2005). High scores for consequences and causes were related to a greater likelihood of taking part in psychotherapy. High control was related to receipt of medication. Finally, psychosocial and biological causes were positively related to medication underuse.

Lastly, four studies have explored the relationship between treatment outcomes and illness representations in adolescents (Munson et al., 2009, 2010; Emilsson et al., 2017; Wong et al., 2019). For adolescents with mood disorders, treatment control was positively related to help seeking (Munson et al., 2009), and a high level of consequences was associated with high adherence (Munson et al., 2010). For adolescents with ADHD, high levels of emotional representations and consequences were linked to high unintentional non-adherence (Emilsson et al., 2017). Finally, low personal control and high emotional representations were related to high adherence to behavioral therapy (Wong et al., 2019).

## Discussion

Understanding the determinants of the psychosocial adjustment and adherence of people with mental disorders is a major health issue. The current review has shown that illness representations are related to both psychosocial adjustment and treatment outcomes in people with mental disorders. More specifically, in adulthood, illness representations are associated with adherence to medication and psychotherapies (for bipolar disorders, psychotic disorders, depression, mixed mental disorders, and PTSD). In addition, illness representations are associated with the individual’s attitude toward medications, such as the need to take them, their harmfulness, as well as distrust of medicine (for psychotic disorders, depression, and mixed mental disorders). The few studies conducted among adolescents have shown similar patterns and have highlighted that illness representations are linked to adherence (for ADHD and mood disorders) and propensity to seek help (for mood disorders). Thus, illness representations play an essential role in adherence and in the treatment decision-making process.

Furthermore, concerning associations between illness representations and psychosocial adjustment, in adulthood, illness representations are associated with symptoms (depressive symptoms, anxiety symptoms, positive and negative symptoms, shape concern, dietary restraint, and suicidal ideations). In psychotic disorders, illness representations are related to quality of life, self-esteem, self-rated mental health, global functioning, insight, and recovery. Finally, illness representations are linked to stigma (for mixed mental disorders) and coping (for depression and bipolar disorder). The few studies conducted with adolescents have shown similar patterns and have highlighted that illness representations are related to stigma (for mood disorders and affective and disruptive disorders), coping (for ADHD), quality of life (for ADHD), and self-esteem (for various mental disorders).

The associations between illness representations, psychosocial adjustment, and treatment outcomes in people with mental disorders indicate that illness representations should be more focused on clinical practice. Specifically, Marcus et al. (2014) recommend targeting illness perceptions (particularly controllability) in the early stages of cognitive behavioral therapy in order to improve engagement and, therefore, outcomes. To change illness representations, psychoeducation seems to be a promising intervention, increasing control and belief in the effectiveness of treatment, and reducing negative emotions about the disorder (Etain et al., 2018; M’Bailara et al., 2019). If one of the objectives of psychoeducation is to improve patients’ knowledge, these authors specify that it is not the increase in knowledge as a result of psychoeducation that improves outcomes, but rather changes in illness representations. In the field of mental health, the practice guidelines recommend the use of psychoeducation (Connolly and Thase, 2011; Norman et al., 2017), and these data provide new explanations for understanding the effectiveness of psychoeducation.

However, some limitations to the applicability of SRM to mental disorders can be noted. Indeed, it is crucial to emphasize that the SRM was originally created to try to understand concrete somatic illness and not mental disorders. Yet, most studies just apply the SRM to mental disorders, arguing that the associations between illness representations, coping, and outcomes show its applicability to mental disorders (e.g., Kelly et al., 2007; Cabassa et al., 2008; Lu et al., 2014). Nevertheless, there are limitations to the use of the SRM for mental disorders. First, the lack of validity of the measurement tools (e.g., Munson et al., 2009). Most studies use scales designed for somatic disorders, but which have not been validated for people with mental disorders. Second, some authors note the need to study illness representations based on the symptomatology of individuals (e.g., Lobban et al., 2004; Averous et al., 2018). Indeed, some psychiatric symptoms can change people’s cognitions, behaviors, and emotions, which can alter their relationship to reality and to their disorder, and consequently, their illness representations. It is important to note that even for somatic diseases, it is complex to determine whether illness representations should be considered as states or traits (Ogden, 2012). The changing nature of these constructs is a central issue in this model. Third, for mental disorders, it can be difficult to dissociate the symptoms of the mental disorders on the one hand, and representations of illness on the other (Lobban et al., 2004). For example, a low personal control in people with psychosis may refer to both illness representations and the symptoms of the disorder, since psychosis can lead to significant difficulties in managing impulses, behaviors, and thoughts.

Therefore, in future studies, it appears necessary to confirm more rigorously the applicability of SRM to mental disorders or even to propose an SRM adapted to mental disorders. To this end, it would be relevant to include insight in the SRM adapted to mental disorders. Indeed, insight is highly diminished in many mental disorders (Amador et al., 1991; Varga et al., 2006) and plays a major role in adjustment and adherence (Novick et al., 2015). In addition, the role of illness perceptions in positive psychosocial outcomes has been highlighted for chronic somatic illnesses (Hagger and Orbell, 2003) but very little for mental disorders. In the future, it would be interesting to study in more detail the role of illness representations on the well-being of people with mental disorders.

## Author Contributions

PA, EC, and LD conceived the study and drafted the manuscript. All authors agreed to the current form of the manuscript.

## Conflict of Interest

The authors declare that the research was conducted in the absence of any commercial or financial relationships that could be construed as a potential conflict of interest.
