# Supplementary material for: Relationship Between Illness Representations, Psychosocial Adjustment, and Treatment Outcomes in Mental Disorders: A Mini Review
Source: Front Psychol. 2020 Jun 12;11:1167. doi: 10.3389/fpsyg.2020.01167 (PMC7309516; doi:10.3389/fpsyg.2020.01167)
Supplement: Supplementary file 1 [file Table_1.docx]

| Supplemental table. Information about studies reviewed. | | | |
| --- | --- | --- | --- |
| Author(s) | Population (disorders, sample size and age) | Design | Main results |
| Aikens et al. (2008). | Unipolar, nonpsychotic major depressive episode  *N* = 165. *M*age = 34.2. | Cross-sectional | The strong beliefs that depression was caused by chemical imbalance and that symptoms were long lasting predicted the perceived necessity of treatment. Patients’ beliefs that their symptoms were due to bad luck/chance and poor understanding were predictors of greater perceived harmfulness. |
| Averous et al. (2018). | Bipolar disorders  N= 38, *M*age = 42.4 | Cross-sectional | Lower emotional representations and higher treatment control were predictors of better adherence. |
| Beck et al. (2012). | Schizophrenia or schizoaffective disorder *N* = 131. *M*age = 44.40. | Cross-sectional | Necessity to take a treatment perceived was related to higher treatment control and chronicity. More perceived concerns were related to greater consequences and less treatment control. Greater distrust in medicines in general was associated with less treatment control and greater consequences. |
| Broadbent et al. (2008). | Psychotic disorders, personality disorders and high service user  *N* = 203. *M*age = 40.86. | Cross-sectional | Higher control and lower identity were linked to better overall functioning. Higher unmet needs (rated by staff) were related to greater consequences, concern and less personal control. Total need score (rated by patients) and unmet need were related to more consequences, timeline, identity, concern, emotional representations and less control (treatment and personal). More timeline, control (treatment and personal), coherence and less concern were linked to better attitude to treatment. More frequent visits to a general practitioner were liked to higher identity, concern and emotional responses. |
| Cabassa et al. (2008). | Depression  *N* = 339. *M*age _=_ 49.73. | Cross-sectional | Symptom severity was positively correlated with timeline (acute/chronic). |
| Cavelti et al. (2012a). | Schizophrenia or schizoaffective disorder  *N* = 142. *M*age = 44.83. | Cross-sectional | Chronicity and consequences perceived mediated the positive association between depressive symptoms and insight. |
| Cavelti et al. (2012b). | Schizophrenia or schizoaffective disorder  *N* = 128. *M*age = 44.31. | Cross-sectional | Higher treatment control and coherence were associated with higher subjective well-being, while higher consequences and emotional representations were associated with lower subjective well-being. Higher treatment control was associated with a lower level of psychopathology. Lower treatment control but higher consequences and emotional representations were associated with greater concern about prescribed medication. Greater necessity was associated with higher chronicity, cyclicity and treatment control. Finally, higher chronicity, cyclicity, treatment control and emotional representations were associated with higher insight (rated per patients). |
| Chan & Mak (2016). | Depression, schizophrenia, bipolar disorder, alcohol use disorder, drug use disorder, and comorbid diagnoses  *N* = 376. *M*age = 43.32. | Cross-sectional | Perceived (personal) controllability was a protective factor against self-stigma and a predictor of recovery. Patients with greater consequences tended to have a higher level of self-stigma. |
| DeJong et al. (2012). | Bulimia nervosa (BN) or BN-type eating disorder not otherwise specified  *N* = 78. Age range = 17‑51. | Cross-sectional | Higher emotional representations were related to higher dietary restraint, concerns about eating /shape /weight, and anxiety. Greater consequences, symptoms and control were associated to lower BMI. Illness duration was negatively related to treatment control. Lower personal control was related to higher shape concern. The cause most frequently endorsed was emotional state (endorsed by 87,3% of participants). |
| Dodd et al. (2017). | Bipolar disorders  *N* = 87. *M*age = 44. | Cross-sectional | Illness perceptions was the second most important predictor, after depression, for personal recovery. Patients with higher control (treatment and personal) recovered better. Patients with higher consequences, emotional representations, identity, and self-blame recovered less well than others. |
| Elwy et al. (2016). | Depression  *N* = 271 veterans. Age range = 21‑71. | Prospective, observational | Veterans who thought the symptoms of depressed mood were cyclical and caused by family problems, and who felt they had personal control, were less likely to receive guideline-concordant depression treatment. |
| Emilsson et al. (2017). | Attention deficit hyperactivity disorder (ADHD)  *N* = 101. *M*age *=* 15.6. | Cross-sectional | More important consequences were related to greater unintentional nonadherence. Girls’ intentional nonadherence was related to the belief that ADHD is a long-term condition, while in boys, unintentional nonadherence was related to understanding their illness. |
| Etain et al. (2018) | Bipolar disorders  n = 103 completer (*M*age = 42) and n = 56 non-completers (*M*age = N/A) | Longitudinal | Those who had completed psychoeducation had “more adaptative” illness perceptions than others. After psychoeducation, participants had better illness perceptions. Changing in illness perceptions was the only variable involved in modifications of functioning. |
| Fialko et al. (2006). | Schizophrenia, schizoaffective and delusional disorder  *N* = 190. *M*age = 37.6. | Cross-sectional | Higher consequences, chronicity, and lower cure/control were associated with suicidal ideations. Higher chronicity, cure/control, and consequences were significantly related to the psychopathology score. |
| Gómez-de-Regil et al. (2014). | Schizophrenia and other schizophrenia spectrum, psychotic disorder  *N* = 61. *M*age = 35.9. | Cross-sectional | Negative emotional and cognitive representations were linked to poorer quality of life and mediated the effect of residual symptoms on quality of life. |
| Houle et al. (2013). | Depression  *N* = 88. *M*age = 42. | Cross-sectional | Greater consequences and greater endorsement of social attributions as a cause were linked to a preference for psychotherapy. |
| Hou et al. (2010). | Bipolar affective disorder  *N* = 35 divided into 2 groups (adherent: *M*age *=* 52.80; nonadherent: *M*age *=* 40.60). | Cross-sectional | A univariate analysis showed that consequence and timeline (acute/chronic) were associated with nonadherence, but these results did not appear in the multivariate analysis, probably because of the strong relationship between age and illness perceptions. |
| Hunot et al. (2007). | Anxiety/depression disorders; physical or chronic pain  *N* = 178. *Mage* = 40.1. | Longitudinal | After 6 months of follow-up, the illness perceptions of individuals who adhered to antidepressant treatment and those who did not adhere to antidepressant treatment were not significantly different. |
| Kelly et al. (2007). | Depression  *N* = 189. *M*age = 45.19 | Cross-sectional | Higher emotional reaction was decisive in use of maladaptive coping. More consequences were related to less problem-solving. In woman, greater (personal) control was associated with more adaptive coping. |
| Lobban et al. (2004). | Schizophrenia  *N* = 124. *M*age = 38.81. | Cross-sectional & longitudinal | In both cross-sectional and longitudinal analyses, a high level of consequences was one of the strongest predictors of poor outcomes (anxiety, depressive symptoms, quality of life, satisfaction with mental health and functioning). |
| Lobban et al. (2005). | Schizophrenia or other psychotic disorder  *N* = 124. *M*age = 38.81 | Cross-sectional & longitudinal | Higher identity, chronicity, cyclicity, consequences, and emotional representations were related to higher positive symptoms. Higher chronicity, consequences and emotional representations, as well as lower perceptions of treatment control, personal control and understanding, were associated to a higher level of negative symptoms. Higher anxiety was associated to higher chronicity, cyclicity, consequences, emotional representations, but lesser treatment control. |
| Lobban et al. (2006). | Schizophrenia  *N* = 49 dyads of patients with a diagnosis of schizophrenia (*M*ag*e* = 35 and one of their close relatives (*M*age = 50) | Cross-sectional | Dyads who had high (vs. low) expressed emotion, had higher discrepancy between relative and patients, firsts had a more negative perception of the illness than the other. |
| Lobban et al. (2013). | Bipolar disorders I and II.  *N* = 91. *M*age = 45 | Longitudinal design with follow-up measures at 12 weeks (T1) and 24 weeks(T2) | Identity, consequences, and personal concern affected time to relapse. Less personal control was related to greater depression. |
| Lu et al. (2014). | Depression or dysthymia  *N* = 110. Age range = 19‑67. | Cross-sectional | More identity, chronicity, consequences and emotional representations, and less (personal and treatment) control were related to emotional outcomes. Less coherence was associated with greater depression. Maladaptive (but not adaptive) rumination mediated the link between illness perception and emotional outcomes. |
| M’Bailara et al. (2019). | Bipolar disorders  N = 78 *M*age = 44.6 | Longitudinal | After educational therapy, patient had better understanding, better (personal and treatment) control, was more in agreement with the diagnosis and had fewer worried and emotional representations. After educational therapy there was no significant change in the subscale of chronicity, severity and consciousness subscales. |
| Maguire et al. (2016). | Schizophrenia  *N* = 71. Age range = 18‑65 | Cross-sectional | Self-rated mental health predicted less personal control and coherence, and more consequences, identity, concern and emotional representations. |
| Marcus et al. (2014). | Schizophrenia spectrum psychosis  *N* = 56. *M*age = 42.4. Divided into 2 equal groups (brief intervention: *M*age = 41.0; control condition) or in a case series. | Longitudinal | The cure/control subscale predicted the beneficial impact of cognitive behavioral therapy on outcomes. |
| Moriarty et al. (2012). | Schizophrenia spectrum psychosis  *N* = 50. Age range = 19–65. | Cross-sectional | Illness perceptions (total score) were not related to activity. |
| Moses (2010). | Affective and disruptive disorders  *N* = 60 dyads of patients (*M*age *=* 14.8) and parents or guardians (*M*age *=* 44.3) | Cross-sectional | Less control and greater chronicity were associated with greater self-stigma. |
| Moses (2015). | Depression and other mental disorders not specified by authors  *N* = 102. Age range = 13–18). | Cross-sectional | Higher control was related to better perception of the benefits of hospitalization and improved self-esteem. Less chronicity *(“expected short term”* in the text*)* was related to higher self-esteem. |
| Munson et al. (2009). | Mood disorders  *N* = 70. *M*age = 15. | Cross-sectional | High treatment control was associated to greater propensity to seek help. Higher emotional representations were associated to more perceived stigma. |
| Munson et al. (2010). | Mood disorders  *N* = 70. *M*age = 15. | Cross-sectional | The fully adherent group perceived more consequences than the non-fully adherent group. |
| O’Mahen et al. (2009). | Perinatal depression  *N* = 82. Age range = 19‑39. | Cross-sectional | Controlling for the level of depression, the perception of chronicity (timeline) was a predictor of treatment use. |
| Oflaz et al. (2015). | Bipolar disorders  *N* = 78 divided into 2 groups (dropout: *M*ag*e =* 37, attendant: *M*age = 35.32). | Cross-sectional | The dropout group perceived fewer consequences, negative emotions, and personal control than the attendant group. |
| Peay et al. (2013). | Bipolar disorders  *N* = 266 parents. Age range = 18–65. | Cross-sectional | Higher use of active/social support was related to perceptions of less severe illness, while self-blame/denial coping was related to perceptions of more severe disorder. Type of coping mediated the relationship between perception of illness severity and adaptation. |
| Peay et al. (2014). | Bipolar disorders  *N* = 266 parents. Median age = 41‑45. | Cross-sectional | Perceived illness severity, measured with the B-IPQ, was not related to parents' ability to cope with the risk of mood disorders in children. |
| Quiles Marcos et al. (2009). | Eating disorders  *n* = 98 patients (*M*age = 20.8). *n* = 68 relatives (*M*age *=* 45). | Cross-sectional | When patients and their relatives perceived a higher level of (treatment and personal) control, patients adapted better. Conversely, when patients and their relatives had high consequences, emotional representations, timeline, cyclical timeline and identity scores, patients had poorer adjustment. |
| Reich et al. (2015). | Depressive disorder, somatoform disorder, and/or adjustment disorder  *N* = 100 into 2 groups (with migration background: *M*age = 46.4, without migration background: *M*age = 45.9) | Cross-sectional | Immigrant inpatients felt greater impairment and less control and cited more fatalistic or supernatural causes. Migration background and illness perception predicted motivation for psychotherapy. Migration background and psychotherapy motivation were mediated by illness beliefs. |
| Rungruangsiripan et al. (2011). | Schizophrenia  *N* = 225. *M*age *=* 37.12. | Cross-sectional | Experience medication side effects and therapeutic alliance had direct impact on illness representations. Then, illness representations directly impacted intention to change. |
| Spoont et al. (2005). | Posttraumatic stress disorder (PTSD)  *N* = 87. *M*age = 61.6. | Cross-sectional | Higher negative impact and most frequently endorsed psychosocial model were related to greater participation in psychotherapy. Greater controllability was related to greater self-medication with prescription drugs, and greater endorsement of psychosocial model was associated to greater self-medication with alcohol or illicit drugs and medication underuse. |
| Stainsby et al. (2010). | Schizophrenia, schizoaffective disorder or depression with psychosis.  *N* = 50. *M*age = 41. | Longitudinal | Higher consequences and less coherence were related to poorer quality of life but did not appear to be significant predictors of outcomes. |
| Theodore et al. (2012). | Psychotic illness  *N* = 81 *M*age = 26. | Cross-sectional | Higher treatment control and fewer consequences were related to better quality of life. Higher emotional representations were related to a lower quality of life. Treatment control helped to explain quality of life. |
| Vanheusden et al. (2009). | Mental health problem.  *N* = 830 Age range = 19‑32. | Cross-sectional | Independently of the sociographic and psychopathological variable, multivariate logical regression showed that higher levels of consequences, treatment control, and intrapsychic causes were associated to an increased likelihood of service use. |
| Ward & Heidrich (2009). | Mental illness  *N* = 185. Age range = 25‑85. | Cross-sectional | Participants who attributed more of their symptoms to the illness and had higher treatment control perceived less stigma. |
| 45. Watson et al. (2006). | Schizophrenia, schizoaffective psychosis and delusional disorder.  *N* = 100. *M*age = 39.1 | Cross-sectional | More chronicity, consequences, symptoms and less cure/control were related to more depression, anxiety and lower self-esteem. Illness perceptions accounted for 46%, 36% and 34% of variance of the depression, anxiety and self-esteem. Depression and perception consequences were dimensions most strongly associated with adherence to medication. |
| Williams & Steer (2011). | Schizophrenia, schizoaffective psychosis, dissociative psychosis, bipolar disorder, affective disorder.  *N* = 66. *M*age = 34.06. | Cross-sectional | Lower consequences, higher perception of (personal and treatment) control, and coherence were linked to better reported engagement. Coherence and treatment control were the strongest predictors of engagement with service. |
| Wong et al. (2019) | ADHD.  N = 63. *M*age = 14.28. | Cross-sectional | A lower “impact” (i.e. the combinaison of several illness representations: consequences, identity, concern and emotional representation items) perceived and belief that “psychological or environmental causes” caused ADHD were predictors of better quality of life. Greater (personal) control, timeline, coherence perceived, and less perceived impact and gender predicted more use of minimization. Lesser perception of coherence and higher perceived impact predicted “reactive-wishful coping” (i.e. combinaison of two coping strategies “emotional reaction” and “wishful thinking”). Illness representations were not predictors of adherence. |

*Notes.* *M*age = mean age; BMI: body mass index; ADHD: attention deficit hyperactivity disorder; N/A: Non available; B-IPQ: Brief Illness Perception Questionnaire.
